# An explainable deep learning model classifies eight categories of pigmented skin lesions on clinical photographs: A multicenter retrospective internal validation study in Japan

**DOI:** 10.1016/j.jdin.2026.07.002

**Published:** 2026-07-23

**Authors:** Kimi Iinuma, Kazuyasu Fujii, Chisa Nakashima, Kenichiro Kasai, Hiroyuki Irie, Hitonari Kanetomo, Shigeto Yanagihara, Sayuri Sato, Hisashi Uhara, Fumiaki Takeda, Yuichi Kimura, Takashi Nagaoka, Atsushi Otsuka

**Affiliations:** a Department of Dermatology, Kindai University Hospital, Sakai, Japan; b Kasai Clinic for Plastic Surgery, Osaka, Japan; c Department of Dermatology, Graduate School of Medicine, Kyoto University, Kyoto, Japan; d Kanetomo Dermatology Clinic, Osaka, Japan; e Department of Dermatology, Sapporo Medical University School of Medicine, Sapporo, Japan; f Hiroshima University Digital Monozukuri (Manufacturing) Education and Research Center, Hiroshima, Japan; g Faculty of Informatics, Cyber Informatics Research Institute, Kindai University, Osaka, Japan; h Faculty of Biology-Oriented Science and Technology, Kindai University, Osaka, Japan

**Keywords:** artificial intelligence, clinical photographs, deep learning, explainability, melanoma, pigmented skin lesions

Pigmented skin lesions are commonly encountered in dermatologic practice, yet their differentiation based on clinical photographs remains challenging, particularly when dermoscopy is unavailable.^1^ We developed an explainable deep learning–based classification model for common pigmented lesions using routine clinical photographs, building upon recent advances in artificial intelligence–based skin lesion classification.^2^

In this multicenter retrospective study, 979 clinical photographs across 8 diagnostic categories (acquired dermal melanocytosis, basal cell carcinoma, ephelis, malignant melanoma, melasma, nevus, seborrheic keratosis, and solar lentigo) were collected from dermatologic institutions in Japan. Benign lesions were diagnosed in routine clinical practice by board-certified dermatologists using established criteria, whereas malignant melanoma and basal cell carcinoma were histopathologically confirmed. The model incorporated architectures pretrained on publicly available dermatologic image data sets.^3^ Data were split at the patient level into training, validation, and independent test sets to prevent data leakage, with each patient assigned to a single subset.

Class-specific receiver operating characteristic and precision–recall curves are shown in (Supplementary Fig 1, available via Mendeley at https://doi.org/10.17632/ndg49xmgpr.1). On the independent test set, the model correctly classified 188 of 196 images, yielding an accuracy of 95.9% (95% CI, 92.1%-98.2%). Melanoma sensitivity was 100% in this limited test set (20/20; 95% CI, 83.2%-100.0%), but this was not powered to establish melanoma safety. Most misclassifications occurred between clinically similar lesions, particularly seborrheic keratosis and basal cell carcinoma (Fig 1). These error patterns reflect known diagnostic challenges among clinically overlapping lesions.

**Fig 1:**
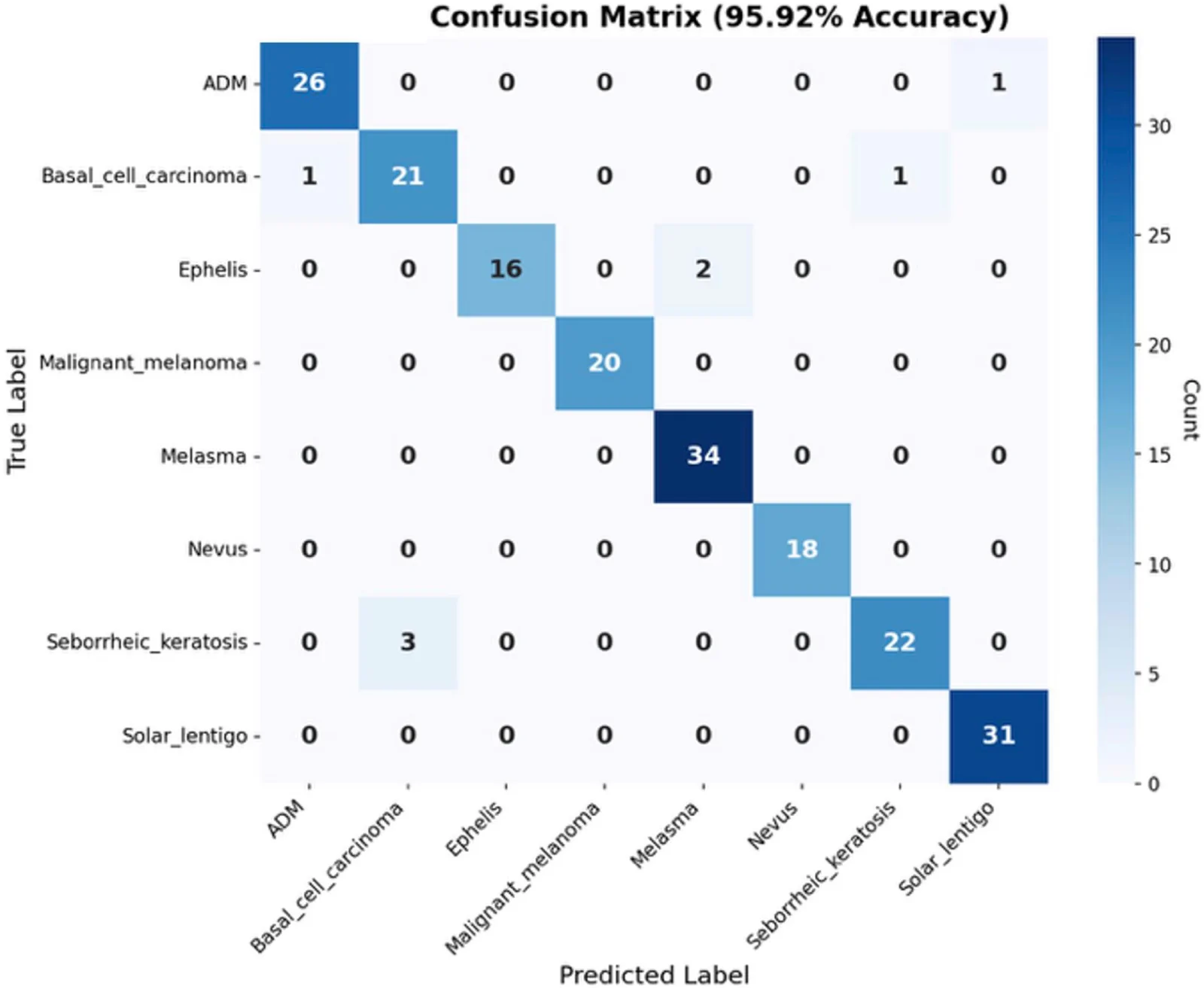
Confusion matrix for the independent test set. Most misclassifications occurred between clinically similar lesions, particularly seborrheic keratosis and basal cell carcinoma. No malignant melanoma cases in this data set were misclassified as benign lesions.

To improve interpretability, Gradient-weighted Class Activation Mapping was applied to convolutional neural network components of the model.^4^ Attention maps demonstrated lesion-centered and disease-specific patterns across categories (Supplementary Fig 2, available via Mendeley at https://doi.org/10.17632/ndg49xmgpr.1). For example, malignant melanoma tended to show relatively concentrated central attention, whereas melasma demonstrated more diffuse activation. These findings are consistent with known dermatologic morphology and suggest that model predictions were primarily driven by lesion-relevant regions, which may help contextualize model attention patterns.^5^ Representative misclassified cases described above are shown in (Supplementary Fig 3, available via Mendeley at https://doi.org/10.17632/ndg49xmgpr.1). However, transformer-based components were not evaluated with explainability methods, and dermatologist assessment of clinical relevance was not performed.

This study should be interpreted as a model development and internal validation study. Although performance metrics were high, the test set was relatively small, particularly for malignant melanoma, and confidence intervals remain wide. The absence of false-negative melanoma cases in this data set should therefore be interpreted cautiously and does not establish melanoma safety. In addition, benign lesions were diagnosed clinically without uniform histopathologic confirmation, which may introduce label uncertainty. The data set was derived from Japanese institutions, which may limit generalizability to other populations, imaging conditions, and skin types.

Despite these limitations, the model demonstrated high internal performance using routine clinical photographs and provided visually interpretable outputs that may assist lesion evaluation. Rather than replacing clinical judgment, such systems may serve as adjunctive tools to assist in the evaluation of pigmented lesions, particularly in settings in which dermoscopy is unavailable. External validation in independent and diverse data sets will be necessary to assess generalizability and clinical utility.

### Declaration of generative AI and AI-assisted technologies in the writing process

During the preparation of this work, the authors used ChatGPT (OpenAI) to assist with language editing. After using this tool, the authors reviewed and edited the content as needed and take full responsibility for the content of the publication.

### Data availability statement

The data that support the findings of this study are available from the corresponding author upon reasonable request. Due to institutional and ethical restrictions, the data are not publicly available.

## Conflicts of interest

None disclosed.
